# Multifaceted role of serine hydroxymethyltransferase in health and disease

**DOI:** 10.1016/j.mocell.2025.100262

**Published:** 2025-07-28

**Authors:** Jing Zhang, Seong Eun Lee, Jiyeon Yoon, Bon Jeong Ku, Junyoung O. Park, Da Hyun Kang, Jun Young Heo, Yea Eun Kang

**Affiliations:** 1 Research Center for Endocrine and Metabolic Disease, Integrated Disease Research Institute, College of Medicine, Chungnam National University, Daejeon, Republic of Korea; 2 Department of Medical Science, Chungnam National University School of Medicine, Daejeon, Republic of Korea; 3 Department of Internal Medicine, Chungnam National University Hospital & College of Medicine, Daejeon, Republic of Korea; 4 Department of Chemical and Biomolecular Engineering, University of California, Los Angeles (UCLA), Los Angeles, CA, USA; 5 Department of Biochemistry, College of Medicine, Chungnam National University, Daejeon, Republic of Korea; 6 System Network Inflammation Control Research center, Chungnam National University, Daejeon, Republic of Korea

**Keywords:** Cancer, Metabolic disease, Neurological disorders, One-carbon metabolism, Serine hydroxymethyltransferase

## Abstract

Serine hydroxymethyltransferase (SHMT) is a key enzyme in 1-carbon metabolism, a biochemical pathway critical for cellular growth, proliferation, and survival. One-carbon metabolism integrates the folate and methionine cycles to produce essential intermediates necessary for nucleotide synthesis, methylation reactions, and redox homeostasis. SHMT exists in 2 isoforms, SHMT1, which is localized in the cytoplasm, and SHMT2, which is localized in the mitochondria. SHMT1 and SHMT2 have distinct yet complementary functions. Both are involved in serine and glycine metabolism, ensuring a continuous supply of the 1-carbon units required for biosynthetic and epigenetic processes. SHMT dysregulation has been implicated in cancer progression and metabolic disorders, including cardiovascular diseases, diabetes, and neurological abnormalities. In cancer, the abnormal expression of SHMT has been associated with tumor growth, metabolic reprogramming, and treatment resistance, and has also been shown to correlate with poor patient outcomes. Considering its critical role in both cancer and metabolic diseases, SHMT has emerged as a potential therapeutic target in cancer. Recent studies have shown that SHMT inhibitors can reduce tumor proliferation and restore metabolic homeostasis. This review provides a comprehensive overview of the role of SHMT in the regulation of metabolic pathways and its role in tumor progression and metabolic diseases. In this review, we aimed to highlight the therapeutic potential of targeting SHMT and offer insights into the development of innovative treatment strategies in oncology and metabolic medicine. These insights support the hypothesis that targeting SHMT, particularly isoform-specific inhibition, may provide novel therapeutic avenues in both oncology and metabolic medicine.

## INTRODUCTION

Metabolism refers to the biochemical processes through which cells and organisms convert food-derived energy (Martínez-Reyes and Chandel, 2020). These processes are dysregulated in cancer and metabolic disorders, leading to abnormal energy production patterns (Lee, 2024). Foundational discoveries in cancer metabolism have revealed metabolic pathways that facilitate rapid cell growth, including aerobic glycolysis (Warburg effect), glutaminolysis, and enhanced nucleotide production (Lee and Kim, 2016, Kim et al., 2024). Among these pathways, one-carbon metabolism (OCM) serves as a central hub by integrating the folate and methionine cycles, supplying critical intermediates for nucleotide biosynthesis, methylation reactions, and redox balance (Locasale, 2013). Owing to its role in maintaining biosynthetic and epigenetic integrity, OCM is critical for rapidly dividing cells, particularly cancer cells, which require a continuous supply of nucleotides and methyl donors to maintain high DNA replication rates and epigenetic modifications (Ducker and Rabinowitz, 2017). Dysregulation of OCM is closely associated with tumorigenesis and cancer progression, making it a prominent target for cancer metabolism (Locasale, 2013).

**Fig. 1:**
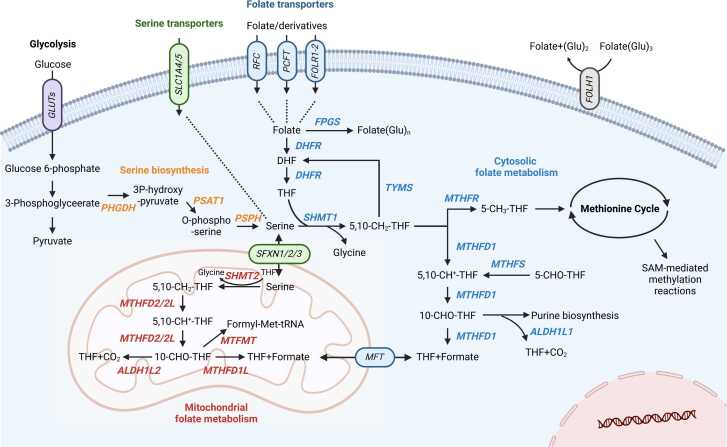
Key enzymes and transporters involved in OCM. Schematic diagram represents the major enzymes and transporters that facilitate OCM and their integration with serine biosynthesis and folate metabolism (created using biorender.com). Glucose undergoes glycolysis to produce 3-phosphoglycerate, which serves as a precursor for serine biosynthesis, and is catalyzed by PHGDH, PSAT1, and PSPH. Serine enters cells via the transporter SLC1A4/5, whereas folate and its derivatives are imported folate transporters. In the cytosol, folate is converted to tetrahydrofolate (THF) by DHFR and further processed by enzymes such as SHMT2, TYMS, and MTHFR to support OCM, which drives nucleotide biosynthesis, methionine cycling, and SAM-mediated methylation reactions. In the mitochondria, SHMT2 facilitates the conversion of serine into glycine and 5,10-CH_2_-THF, which are utilized in OCM by methylenetetrahydrofolate dehydrogenase 2/2L and MTHFD1L to generate formate and other intermediates that integrate back into cytosolic folate metabolism. Key transporters, such as SFXN1/2/3, enable the mitochondrial import of serine and glycine, whereas MFT facilitates the exchange of formate and THF between the mitochondria and the cytosol. This interconnected network underscores the essential roles of serine and folate metabolism in cellular processes such as nucleotide biosynthesis, methylation reactions, and maintenance of redox homeostasis.

Serine hydroxymethyltransferase (SHMT) is a key enzyme in OCM with 2 major isoforms: SHMT1 in the cytoplasm and SHMT2 in the mitochondria (Ducker and Rabinowitz, 2017). SHMT1 and SHMT2 participate in distinct steps of the folate cycle (Tibbetts and Appling, 2010). SHMT1 converts serine and tetrahydrofolate (THF) into glycine and 5,10-methylene-THF (5,10-CH_2_-THF) in the cytoplasm, producing essential intermediates for thymidylate and purine biosynthesis (Yang and Vousden, 2016). It also plays a particularly crucial role in DNA replication and the rapid proliferation of tumor cells through its function in thymidylate synthesis (Zhang and Wang, 2024). Conversely, SHMT2 functions in the mitochondria, where it converts serine into glycine and generates 5,10-CH_2_-THF (Zeng et al., 2021). This process supports mitochondrial protein synthesis and enhances cell survival (Meiser et al., 2018). The combined functions of SHMT1 and SHMT2 ensure a steady supply of 1-carbon units necessary for nucleotide biosynthesis and mitochondrial redox homeostasis (Anderson and Stover, 2009). SHMT1 acts in the cytoplasm and regulates the expression of SHMT2 by binding to the 5′ nontranslating region of SHMT2 through RNA-binding activity (Guiducci et al., 2019). Thus, SHMT1 indirectly regulates the expression of mitochondrial SHMT2 and balances the flow of 1-carbon units between the cytoplasm and mitochondria. The importance of SHMT in intracellular metabolism is linked to various diseases, specifically cancer and metabolic disorders; however, it still needs to be comprehensively understood in different cancer types and organs (Fig. 1).

In this review, we provide a comprehensive analysis of the dual roles of SHMT in health and disease, focusing on its roles in cancer metabolism, metabolic disorders, and its therapeutic potential. By synthesizing the recent advances in SHMT research, we aimed to emphasize its metabolic and regulatory functions and offer insights into the development of novel therapeutic strategies for several diseases.

## ROLE OF SHMT IN OCM

OCM is an important biochemical process for maintaining normal cellular functions and primarily involves the transfer and utilization of 1-carbon units (Ducker and Rabinowitz, 2017). Its key components are the folate and methionine cycles. SHMT serves as a central enzyme in the OCM, catalyzing the transformation of serine into glycine and providing essential 1-carbon units (Yang and Vousden, 2016).

### Folate Cycle

The folate cycle forms the foundation of OCM (Ducker and Rabinowitz, 2017). It primarily involves THF, the active form of folate, which serves as a carrier for 1-carbon unit transfer and utilization (Locasale, 2013). SHMT plays a critical role in the folate cycle and exists as 2 isoforms: SHMT1 in the cytoplasm and SHMT2 in the mitochondria (Ducker and Rabinowitz, 2017). Dietary folate is transported into the cells and reduced to THF (Zhao et al., 2011). In the cytoplasm, SHMT1 catalyzes the reaction between THF and serine to produce 5,10-CH_2_-THF and glycine (Yang and Vousden, 2016). 5,10-CH_2_-THF supports thymidylate and purine biosynthesis by supplying the essential precursors for DNA replication and repair (Dekhne et al., 2020a). Mitochondrial SHMT2 catalyzes a parallel reaction that produces glycine and 5,10-CH_2_-THF within the mitochondria (Minton et al., 2018). The 5,10-CH_2_-THF generated in the mitochondria can not only be exported to the cytoplasm for further metabolism (Konno et al., 2017), but can also be oxidized to 10-formyl-THF by methylenetetrahydrofolate dehydrogenase 2 (Shin et al., 2014). 10-formyl-THF is essential for the initiation of mitochondrial protein translation (Cuthbertson et al., 2021). SHMT1 functions in the cytoplasm and modulates SHMT2 expression by binding to the 5′ nontranslating region of SHMT2 through its RNA-binding activity (Guiducci et al., 2019). Consequently, SHMT1 and SHMT2 coordinate the folate cycle and contribute to cellular proliferation and metabolic demands in specific contexts such as lung cancer. In lung cancer, SHMT1 indirectly influences mitochondrial SHMT2 expression and helps maintain a balance in the 1-carbon unit flux between the cytoplasm and mitochondria (Guiducci et al., 2019). However, further studies are needed to determine whether this regulatory mechanism is conserved across other cancer types.

### Methionine Cycle

The methionine cycle is another vital component of the OCM (Ducker and Rabinowitz, 2017). It is primarily responsible for generating the universal methyl donor S-adenosylmethionine (SAM) and recycling homocysteine to sustain methylation reactions and metabolic homeostasis (Mentch and Locasale, 2016). Although SHMT does not participate directly in the methionine cycle, its indirect contribution is essential for proper functioning of the methionine cycle. Under the catalytic action of SHMT1 in the cytoplasm, THF is converted to 5,10-CH_2_-THF (Paone et al., 2014). Then, the 5,10-CH_2_-THF is reduced to 5-methyl-THF (5-CH_3_-THF) by methylenetetrahydrofolate reductase (MTHFR), which provides the methyl group required for methionine regeneration (Pinweha et al., 2016).

## SHMT IN METABOLIC DISEASE

### SHMT in Metabolic Dysfunction-Associated Steatotic Liver Disease (MASLD)

Recent studies have highlighted the multifaceted roles of SHMT in liver metabolism, particularly in terms of lipid metabolism, the oxidative stress response, and its implications in liver diseases (Anderson and Stover, 2009, Cuthbertson et al., 2021). In hepatic lipid metabolism, SHMT1 regulates hepatic lipid metabolism by modulating SAM levels, which influences lipid methylation and indirectly affects hepatic lipid deposition (Zhang et al., 2021). In OCM, SHMT1 catalyzes the reversible conversion of serine to glycine to generate 5,10-CH_2_-THF (Yang and Vousden, 2016). Its downstream product SAM serves as a crucial methyl donor in various methylation reactions, including those involved in lipid metabolism (Cuthbertson et al., 2021). SHMT2 has been shown to modulate lipid accumulation through glycine-mediated activation of the mTOR pathway. Silencing SHMT2 in hepatocytes led to reduced lipid accumulation, accompanied by downregulation of the mTOR/PPARγ pathway. This effect is attributed to decreased glycine levels, suggesting that SHMT2 influences lipid metabolism via glycine-dependent mTOR activation (Choi et al., 2022).

SHMT1 and SHMT2 play crucial roles in the pathogenesis of MASLD. DNA damage and chromosomal instability are common features of MASLD (Barchetta 2025 ). SHMT1 plays a crucial role in decline in thymidylate (dTMP) synthesis and maintenance of DNA stability. Previous studies indicated that SHMT1 deficiency impairs thymidylate synthesis, increases DNA damage, and leads to abnormal DNA methylation. Moreover, genetic polymorphisms in SHMT1, such as the L474F mutation, may affect SUMOylation and nuclear localization, thereby influencing genome stability (Anderson and Stover, 2009). Therefore, we speculate that SHMT1 deficiency may increase the risk of MASLD progression to liver fibrosis and even hepatocellular carcinoma (HCC). SHMT2 also influences MASLD pathogenesis by regulating glycine levels. In steatotic livers, the increased synthesis of serine from glycine via reverse SHMT activity leads to decreased glycine levels. This reduction in glycine levels impairs glutathione synthesis, making the liver more susceptible to oxidative stress and acetaminophen-induced hepatotoxicity. These findings suggest that targeting SHMT2 to modulate glycine levels may be a potential therapeutic strategy for MASLD and other hepatic disorders (Ghrayeb et al., 2024, McBride et al., 2024).

In addition, disturbances in SHMT2 activity have been linked to broader metabolic imbalance. For example, increased serine synthesis from glycine via reverse SHMT2 activity contributes to glycine depletion in steatotic liver. This leads to reduced glutathione synthesis and increased oxidative damage. Given the liver’s central role in lipid metabolism, inflammation, and systemic homeostasis, SHMT2 dysfunction may also exacerbate extrahepatic complications, such as metabolic syndrome and cardiovascular comorbidities (Ghrayeb et al., 2024).

The role of SHMT in liver inflammation and fibrosis has also been investigated. There is currently no direct evidence that SHMT1 affects MASLD-related inflammation or fibrosis. However, limited studies in other cell types suggest that SHMT1 may influence inflammatory signaling pathways such as NF-κB, which plays a key role in chronic inflammation associated with MASLD. Moreover, sustained activation of NF-κB promotes the release of proinflammatory cytokines, exacerbates hepatocyte damage, and accelerates fibrosis progression (Zhang et al., 2023). Although the involvement of SHMT1 in hepatic immune regulation remains unconfirmed, it is possible that SHMT1 deficiency or dysfunction may influence MASLD-related inflammatory responses through the NF-κB pathway, thereby contributing to disease progression from steatosis to liver fibrosis or even HCC. This hypothesis warrants further investigation. However, SHMT2’s involvement in these pathological processes has been established. Studies have indicated that while SHMT2 deficiency can reduce fatty liver, it may exacerbate liver inflammation and fibrosis under certain conditions. This highlights the complex role of SHMT2 in liver pathology, where its activity may have both protective and detrimental effects, depending on the context. SHMT2 is involved in oxidative stress regulation and cellular reprogramming, further emphasizing its critical function in liver health and pathology, suggesting that targeting SHMT2 could offer therapeutic potential for a range of liver diseases (Chen et al., 2024, Wang et al., 2019).

In addition to its role in liver metabolism and oxidative stress, recent evidence suggests that lipid metabolic dysregulation in MASLD may create a prometastatic hepatic microenvironment. Specifically, altered lipid metabolism affects immune surveillance and promotes an immunosuppressive niche in the liver, thereby predisposing MASLD patients to hepatic metastasis (Kim and Hyun, 2024). This underscores the importance of lipid metabolic pathways not only in the progression of MASLD itself but also in its potential to facilitate secondary tumor seeding in the liver.

Collectively, these studies underscore the significance of SHMT in liver metabolism through its influence on lipid accumulation, oxidative stress responses, and cancer cell proliferation. Unlike SHMT1, whose role in MASLD-related inflammation and fibrosis remains unclear, SHMT2 has been more clearly implicated in metabolic dysfunction-associated liver disease (Ghrayeb et al., 2024, McBride et al., 2024, Zhang et al., 2023). Its involvement in critical metabolic pathways makes it a potential therapeutic target for various liver diseases, including MASLD and HCC. Future research focusing on the precise mechanisms by which SHMT2 regulates these processes is essential for developing targeted therapies to modulating its activity in liver pathology.

### SHMT in Metabolic Syndrome and Obesity

The role of SHMT in metabolic disorders, including metabolic syndrome and obesity, highlights its potential as a therapeutic target (Chen et al., 2024, Liu et al., 2023).

The role of SHMT in hepatic function has garnered significant attention in the context of metabolic syndrome and obesity. Studies have shown that SHMT1 inhibits NADPH oxidase 1 in HCC, thereby reducing ROS levels, which in turn suppresses tumor cell invasion and metastasis, indicating that SHMT1 plays a critical role in maintaining metabolic homeostasis (Dou et al., 2019). SHMT2 plays a pivotal role in hepatic function and metabolic stability. A previous study demonstrated that the liver-specific deletion of Shmt2 in mice led to alterations in amino acid levels, particularly a significant deficiency of 1-carbon units in the liver. This disruption impairs the hepatic methylation potential, which is crucial for lipid metabolism and overall liver function, potentially contributing to the development of metabolic disorders (Chen et al., 2024). Recent studies have explored how disturbances in the mitochondrial OCM, including impaired SHMT2 activity, can lead to global DNA methylation changes and metabolic imbalances. For example, metformin was shown to regulate DNA methylation through mitochondrial OCM in an SHMT2-dependent manner (Cuyas et al., 2018). These epigenetic and metabolic alterations have been implicated in the development of obesity and other metabolic disorders.

In contrast, SHMT1 and SHMT2 influence adipose tissue methylation and energy metabolism via distinct mechanisms during the development of obesity.

SHMT1 modulates DNA methylation in adipose tissue by regulating the partitioning of 1-carbon units between dTMP and SAM synthesis. Notably, SHMT1-deficient mice exhibit NTDs under conditions of maternal folate deficiency, a mechanism linked to DNA damage caused by impaired thymidylate synthesis (Beaudin et al., 2011). These metabolic defects may increase the risk of obesity later in life through epigenetic regulation (Wang et al., 2019, Xie et al., 2016). Furthermore, SHMT1 competitively consumes 5-CH₃-THF, thereby inhibiting SAM-dependent methylation reactions, regulating the expression of adipogenesis-related genes such as PPARγ, and ultimately influencing adipocyte differentiation (Herbig et al., 2002).

SHMT2 has been implicated in the regulation of glycine levels, which are often decreased in obesity and associated metabolic disorders. Glycine plays a critical role in various metabolic pathways, including the synthesis of glutathione, a major antioxidant. Altered glycine metabolism can exacerbate oxidative stress and inflammation, common features of obesity-related conditions. Recent studies have highlighted the importance of diet, gut microbiota, and liver metabolism in determining glycine availability in obesity, suggesting that enhancing glycine levels may be beneficial for mitigating metabolic disturbances (Alves et al., 2019, Liufu et al., 2024).

The involvement of SHMT2 in apoptosis has been explored in the context of metabolic health. Research has indicated that SHMT2 depletion induces apoptosis through lysosomal membrane permeabilization, which is mediated by excessive activation of the autophagy-lysosome pathway via metabolic reprogramming. This finding suggests that SHMT2 is essential for cell survival under metabolic stress conditions, which are prevalent in obesity and metabolic syndromes (Liu et al., 2023).

Moreover, the disruption of SHMT2 function has been linked to developmental anomalies that may predispose individuals to metabolic disorders. A previous study reported that disruption of Shmt2 in mice resulted in embryonic anemia due to impaired erythropoiesis, indicating a SHMT2 critical role in early development. Although this study focused on embryonic development, it underscores the importance of SHMT2 in cellular proliferation and differentiation, processes that are often dysregulated in metabolic diseases (Tani et al., 2019).

In summary, SHMT is integral to the maintenance of metabolic homeostasis. SHMT1 primarily regulates DNA methylation, adipocyte differentiation, and inflammation, whereas SHMT2 is mainly involved in mitochondrial OCM, oxidative stress resistance, and cell survival, with significant effects on hepatic function and glycine metabolism (Alves et al., 2019, Dou et al., 2019, Liufu et al., 2024). Alterations in SHMT activity can disrupt these processes and contribute to the pathogenesis of metabolic syndrome and obesity. Further research is warranted to fully elucidate SHMT2 role and to explore its potential as a therapeutic target for metabolic disorders.

## SHMT IN CARDIOVASCULAR DISEASES

Although the importance of SHMT in cellular metabolism is well-established, direct evidence linking SHMT to cardiovascular disease remains sparse. However, its known functions and involvement in related metabolic pathways suggest that SHMT plays a significant role in cardiovascular health, warranting further investigation (Boehme et al., 2018, Garcia-Cazorla et al., 2020). SHMT1 plays a pivotal role in vascular calcification. In vascular smooth muscle cells, SHMT1 silencing induces oxidative stress, increases alkaline phosphatase activity, and upregulates the osteogenic markers MSX2 and CBFA1, thereby exacerbating phosphate-induced vascular calcification (Boehme et al., 2018). Moreover, SHMT1 functions as a metabolic switch that regulates the critical balance in OCM. When SHMT1 is overexpressed, 1-carbon flux is preferentially directed toward dTMP synthesis, leading to a reduction in SAM production, which subsequently alters the DNA methylation status (Herbig et al., 2002). This metabolic shift may contribute to an imbalance in energy metabolism in cardiomyocytes and to pathological cardiac remodeling, suggesting that SHMT1 is a key metabolic regulator in cardiovascular disease.

Given the liver’s role in systemic lipid and glycine homeostasis, SHMT2-associated hepatic dysfunction may have downstream effects on cardiovascular health by promoting systemic inflammation and metabolic stress (Ghrayeb et al., 2024).

Additionally, SHMT2 has been implicated in developmental syndromes that affect both the nervous and cardiac systems. Mutations in SHMT2 have been associated with a novel developmental syndrome characterized by cognitive impairment, motor dysfunction, and progressive heart disease. These findings underscore the enzyme’s importance in the development and function of cardiac tissues and suggest that SHMT2 is essential for proper cardiovascular development and maintenance (Garcia-Cazorla et al., 2020).

Although direct evidence linking SHMT to cardiovascular diseases is still emerging, these studies highlight the potential role of SHMT in cardiovascular health through its involvement in metabolic pathways and developmental processes (Boehme et al., 2018, Ghrayeb et al., 2024). Further research is necessary to elucidate the specific mechanisms by which SHMT influences cardiovascular disease progression and to explore its potential as a therapeutic target.

## SHMT IN NEUROLOGICAL DISORDERS

Recent studies have highlighted the significance of SHMT in neurological disorders, in which its dysfunction can lead to profound neurodevelopmental and neurodegenerative consequences (Abarinov et al., 2013, Garcia-Cazorla et al., 2020).

The precise role of SHMT1 in neurodevelopment remains unclear; however, its primary mechanism is linked to the folate-dependent dTMP biosynthesis pathway, which is crucial for DNA replication, neural tube closure, and proper nervous system development (Abarinov et al., 2013, Martiniova et al., 2015). SHMT1 regulates folate-mediated de novo dTMP synthesis, ensuring stable DNA replication. Studies have shown that SHMT1 knockout mice exhibit a significantly increased incidence of neural tube defects (NTDs) when subjected to low-folate and low-choline diets, highlighting SHMT1 as a critical regulator of folate metabolism during neural tube development (Beaudin et al., 2012, Beaudin et al., 2011, Martiniova et al., 2015). Moreover, SHMT1 expression in the hippocampus, particularly within the granule cell layer, is closely associated with cognitive and memory function in adult mice. SHMT1-deficient mice display impaired hippocampal neurogenesis and memory deficits, further underscoring its essential role in the neurodevelopment and functional maintenance of the nervous system (Abarinov et al., 2013). However, direct evidence indicates that mutations in SHMT2 are linked to novel neurodevelopmental syndromes characterized by cognitive impairment, motor dysfunction, and structural brain abnormalities. Impairment of SHMT2 disrupts mitochondrial OCM, leading to developmental syndromes that affect both the brain and heart. Patients exhibit microcephaly, intellectual disability, and cardiomyopathy, underscoring the role of this enzyme in organ development and function (Garcia-Cazorla et al., 2020). In support of these findings, a subsequent report detailed a neurodevelopmental disorder associated with a novel homozygous missense variant of SHMT2. The affected individual presented mild dysmorphism, intellectual disability, spasticity, peripheral neuropathy, and a thin corpus callosum. This case expands the phenotypic spectrum of SHMT2-related disorders and emphasizes the importance of this enzyme in central nervous system development (Majethia et al., 2022). The critical role of SHMT2 in mitochondrial function underscores its relevance to neurological health. Disruption of the Shmt2 gene in murine models has been shown to cause embryonic anemia due to impaired mitochondrial OCM, highlighting the essential function of this enzyme in cellular energy homeostasis. Although this study focused on hematopoiesis, the findings have broader implications for tissues with high metabolic demands, such as the brain (Escande-Beillard et al., 2020, Pan et al., 2024, Tani et al., 2019).

Beyond the developmental implications, SHMT1 polymorphisms have been associated with an increased risk of various neurological disorders, including NTDs and acute lymphoblastic leukemia. Studies have demonstrated that SHMT1 knockout mice exhibit a higher incidence of NTDs under folate- and choline-deficient dietary conditions (Beaudin et al., 2012). Further investigations revealed that SHMT1 deficiency disrupts dTMP synthesis, subsequently impairing DNA repair and cell division, thereby increasing the risk of NTDs (MacFarlane et al., 2011a). Additionally, the C1420T polymorphism in SHMT1 has been linked to increased susceptibility to childhood acute leukemia, suggesting a broader role for SHMT1 in DNA metabolism-related pathologies (Skibola et al., 2002). Moreover, the involvement of SHMT1 in vascular smooth muscle cell calcification suggests its potential role in vascular neurological diseases, warranting further investigation into its contribution to cerebrovascular pathology (Boehme et al., 2018).

SHMT2 has been implicated in Alzheimer's disease (AD) pathology through its role in enhancing ADAM10 translation via interaction with the 5' untranslated region of ADAM10 mRNA. This study demonstrated that the small molecule kenpaullone increases SHMT2 expression, which in turn promotes ADAM10 translation, potentially leading to amelioration of AD-related pathology in a mouse model (Song et al., 2024).

In summary, SHMT1 primarily regulates folate metabolism, neural tube closure, and cognitive function in neurological disorders, and may indirectly affect neuronal activity by modulating SHMT2 expression and activity (Abarinov et al., 2013, Guiducci et al., 2019). SHMT2 is integral to mitochondrial OCM and its dysfunction is associated with a spectrum of neurological metabolic disorders (Majethia et al., 2022). Ongoing research into SHMT functions and regulatory mechanisms holds promise for the development of targeted therapies aimed at restoring metabolic balance and improving neurological outcomes.

## MULTIFACETED ROLE OF SHMT IN CANCERS

Cancer cells undergo extensive metabolic reprogramming to support rapid proliferation, survival, invasion, and metastasis (Kim and Hyun, 2024, Varli et al., 2024, Lee and Yoon, 2024). In addition to fueling growth, metabolic reprogramming also enables cancer cells to adapt to microenvironmental stresses and support other malignant behaviors (Lee and Kim, 2016). This reprogramming includes alteration of OCM, enhanced nucleotide biosynthesis, dysregulated redox homeostasis, and increased dependency on serine and glycine metabolism to sustain rapid cell growth and adaptation to metabolic stress (Pavlova et al., 2022). OCM plays a crucial role in metabolic reprogramming and supports cancer cell survival (Ducker and Rabinowitz, 2017). As a key enzyme in OCM, SHMT facilitates the synthesis of nucleotides, biosynthetic precursors, and methyl donors that are critical for cancer progression (Yang and Vousden, 2016).

### Overview of SHMT1 and SHMT2 Expression Across Cancer Types

Across various cancer types, SHMT1 and SHMT2 exhibit distinct expression patterns and isoform-specific functional roles.

SHMT1 displays variable expression and diverse functional roles depending on cancer type. It has been shown to act as a tumor suppressor in some contexts, such as HCC, where it mitigates oxidative stress and epithelial-mesenchymal transition (Dou et al., 2019). It is also upregulated in papillary thyroid carcinoma (PTC) and ovarian cancer, where it may play tumor-promoting roles (Gupta et al., 2017, Sun et al., 2016).

Meanwhile, SHMT2 is generally upregulated in more aggressive malignancies and promotes tumor progression by enhancing nucleotide biosynthesis, redox balance, and metabolic flexibility (Cuthbertson et al., 2021, Yang and Vousden, 2016). Large-scale datasets, including bulk RNA-seq data from The Cancer Genome Atlas (TCGA), microarray profiles from the Gene Expression Omnibus (GEO), and clinical cohorts, reveal that SHMT2 overexpression is a common feature in lung, colorectal, thyroid, pancreatic, and ovarian cancers, and is often associated with poor prognosis (Lin et al., 2022, Liu et al., 2019, Noguchi et al., 2018, Wallace-Povirk et al., 2024). However, in prostate cancer, SHMT2 displays a more complex expression pattern, being elevated in early-stage or low-grade tumors but reduced in more advanced cases, suggesting a stage-dependent role (Chen et al., 2022).

These isoform-specific differences highlight the need for context-aware, cancer-type-specific evaluation of SHMTs. Notably, gene expression alone may not fully reflect their biological impact, as post-translational modifications and subcellular localization also influence enzymatic activity. A comparative summary of SHMT expression patterns and functional mechanisms across cancers is presented in Table 1, Table 2, Table 3, Table 4.

**Table 1:** Expression of SHMT in tumor clinical samples

Serine hydroxymethyltransferase	Cancer type	Sample	Expression pattern	Clinical association	Reference
SHMT1	Lung cancer (NSCLC)	NSCLC patients, n = 97; TCGA	High expression (in low-folate NSCLC)	No significant	(Lin et al., 2022)
		GEO	High expression	Unknown	(Paone et al., 2014)
	Lung cancer (SCLC)	GWAS	High expression	SCLC risk (↓)	(Wu et al., 2023)
	Liver cancer (HCC)	HCC patients, n = 120	Low expression	Poor OS	(Dou et al., 2019)
		10 paired cancerous and para- carcinomatous tissues; TCGA	Low expression	Poor OS	(Tang et al., 2024)
		8 HCC patients with confirmed unequivocal HCC diagnosis and homologous cancer-free liver tissue	Low expression	SHMT1 silencing (via promoter methylation) → alcohol-related HCC development	(Udali et al., 2015)
	Thyroid cancer (PTC, PDTC)	Tissue microarray of 344 PTC, 46 PDTC cases	High expression in tumor and stroma (vs MTC and FTC)	Stromal SHMT1 expression in FVPTC → shorter DFS	(Sun et al., 2016)
	Thyroid cancer (ATC)	17 tumor-normal pairs; bulk transcriptomics of 263 normal thyroid, 348 papillary, and 21 undifferentiated thyroid cancer samples; single-cell transcriptomes from 15 cases; TCGA, GEO	Low expression	Positively correlated with TDS	(Lee et al., 2024b)
	Ovarian cancer (HGSOC)	TCGA	High expression	Tumor growth and migration (↑)	(Gupta et al., 2017)
SHMT2	Lung cancer (LUAD)	TCGA, GEO	High expression	Poor prognosis Immune cells: CD4⁺ T cells, macrophages, and dendritic cells (↓)	(Luo et al., 2021)
	Lung cancer (NSCLC)	NSCLC patients, n = 97; TCGA	High expression (in low-folate NSCLC)	Poor prognosis Lower DFS	(Lin et al., 2022)
		GEO	High expression	Unknown	(Paone et al., 2014)
	Lung cancer (SCLC, AC, and SQCLC)	Lung cancer patients, n = 323	High expression	No significant	(Yao et al., 2021)
	Liver cancer (HCC)	HCC patients, n = 144	High expression	Poor OS	(Ji et al., 2019)
		HCC patients, n = 100	High expression	Poor prognosis	(Wu et al., 2016)
	Colorectal cancer	CRC patients, n = 60	High expression	Shorter RFS/DSS Tumor size (↑) LN metastasis (↑) TNM stage (↑) Histological grade (↑)	(Liu et al., 2019)
		CRC patients, n = 43	High expression	Poor OS	(Kim et al., 2023)
		TCGA	High expression	Poor OS and DFS	(Abdelmaksoud et al., 2024)
		309 paired CRC and normal colon specimens	High expression	Poor OS	(Wei et al., 2018)
		CRC patients, n = 85	High expression	Poor OS Advanced T stage LN metastasis	(Liu et al., 2021)
		CRC patients, n = 201	High expression	Poor OS Advanced CRC (stages III and IV)	(Cui et al., 2022)
		CRC patients, n = 5; TCGA, GEO	High expression	Survival time (↓)	(Wang et al., 2022)
		CRC tissues, n = 50, and adjacent normal colon tissues, n = 50; paraffin-embedded samples of stages II and III primary CRC, n = 378; TCGA and GEO	Low expression (high SHMT2 expression in 43.39%)	Low SHMT2 expression + 5-FU → poor OS and DFS	(Chen et al., 2021)
	Prostate cancer	CCLE, TCGA, and GEO	High expression in cancer (vs benign) Low expression in high Gleason score (≥8 vs ≤7)	Tumor aggressiveness (↓)	(Chen et al., 2022)
		Prostate cancer tissues, n = 63	High expression in G6 (vs G9)	Tumor aggressiveness (↓)	(Marrocco et al., 2019)
		16 paired prostate cancer and benign samples; TCGA	High expression	Short DFS	(Sant’Anna-Silva et al., 2021)
		GEO	High expression	Poor OS	(Lyu et al., 2017)
	Thyroid cancer (ATC)	17 tumor-normal pairs; bulk transcriptomics of 263 normal thyroid, 348 papillary, and 21 undifferentiated thyroid cancer samples; single-cell transcriptomes from 15 cases; TCGA, GEO	High expression (vs normal and PTC)	Tumor size (↑) Distant metastasis (↑) TDS (↓)	(Lee et al., 2024b)
	Pancreatic cancer	PDAC patients, n = 103	High expression	Poor OS	(Noguchi et al., 2018)
	Ovarian cancer	TCGA	High expression	Poor prognosis	(Zeng et al., 2021)
		48 lyophilized cDNAs from primary EOC specimens (8 stage I, 9 stage II, 17 stage III, and 6 stage IV) and 8 cDNAs from normal ovaries; TCGA	High expression	Poor prognosis	(Wallace-Povirk et al., 2024)

LUAD, lung adenocarcinoma; NSCLC, non–small-cell lung cancer; SCLC, small-cell lung cancer; AC, pulmonary adenocarcinoma; SQCLC, squamous cell lung cancer; HCC, hepatocellular carcinoma; CRC, colorectal cancer; ATC, anaplastic thyroid carcinoma; PTC, papillary thyroid carcinoma; PDTC, poorly differentiated thyroid carcinoma; FTC, follicular thyroid carcinoma; MTC, medullary thyroid carcinoma; FVPTC, follicular variant PTC; PDAC, pancreatic ductal adenocarcinomas; HGSOC, high-grade serous ovarian cancer; DFS, disease-free survival; OS, overall survival; DSS, disease-specific survival; TCGA, the cancer genome atlas; GEO, gene expression omnibus; GWAS, genome-wide association study; pQTL, protein quantitative trait loci; 5-FU, 5-Fluorouracil; CCLE, cancer cell line encyclopedia; LN, lymph node; RFS, recurrence-free survival; TDS, thyroid differentiation score.

**Table 2:** Role of SHMT protein in various cancers

Serine hydroxymethyltransferase	Cancer type	Role of SHMT	Mechanism	Reference
SHMT1	Lung cancer (NSCLC)	iSHMT1 (cell) → apoptosis (↑)	dTMP (↓), uracil misincorporation	(Paone et al., 2014)
		SHMT1 downregulation (cell) → apoptosis (↑)	Uracil misincorporation, SHMT2 (↑)	(Giardina et al., 2018)
		SHIN1 (SHMT1 inhibitor) +CHIR99021 (mouse) → tumor growth (↓)	OCM (↑)	(He et al., 2022)
		SHMT1 inhibition (cell) → tumor growth (↓)	Viability (↓), doubling time (↓), and tumor growth (↓)	(Lee et al., 2024a)
		SHMT1/2 inhibition (mouse) → tumor growth (↓)	Incorporation of 13C into glycine from [U-13C] serine (↓), apoptosis (↑), and proliferation (↓)	
	Lung cancer (LUAD)	SHMT1 (cell) → sustained proliferation	Controlled cellular SHMT2 mRNA and protein levels by binding to SHMT2 5′UTR sequences in vitro	(Guiducci et al., 2019)
		SHMT1 knockdown (cell) → migration (↓)	Intracellular serine (↓), ATP (↓), ROS (↑), OCR (↑), ECAR (↑), and pThr172AMPK/AMPK (↑)	(Bouzidi et al., 2020)
	Liver cancer (HCC)	SHMT1 (↓) → motility (↓)	Cell metastasis (↓), ROS production (↓), NOX1 expression (↓), EMT production (↓), and MMP2 production (↓)	(Dou et al., 2019)
SHMT2	Lung cancer (NSCLC)	SHIN2 (SHMT2 inhibitor) + paclitaxel (mouse) → tumor growth (↓)	SHIN2 → SHMT2 (↓) → NADPH (↓), ROS (↑) → apoptosis (↑), tumor cell proliferation (↓)	(Lee et al., 2024a)
		↑ SHMT2 under low glucose (low- glucose condition) (cell) → maintained mitochondrial translation and proliferation	Conversion of serine to glycine (↑), providing 1-carbon units for the generation of mitochondrial formylmethionyl-tRNAs	(Haitzmann et al., 2024)
	Lung cancer (LUAD)	pSer90-SHMT2 (cell) → tumorigenesis (↑)	MAPK1 → SHMT2 Ser90 phosphorylation (↑), SHMT2 protein stability (↑), SAM production (↑), and RNA m6A modification (↑)	(Han et al., 2024)
	Lung cancer (SCLC, AC, and SQCLC)	SHMT2 knockdown (cell) → proliferation (↓)	Supports OCM	(Yao et al., 2021)
	Liver cancer (HCC)	siSHMT2 (cell) → proliferation (↓), and invasion (↓)	E-cadherin (↑), glycine uptake (↑), N-cadherin (↓), vimentin (↓), and snail (↓)	(Ji et al., 2019)
		SHMT2 knockdown (cell) → proliferation (↓), migration (↓), and invasion (↓)	Not specified	(Wu et al., 2016)
		SHMT2 knockdown (cell) → proliferation and tumorsphere (↓)	Not specified	(Woo et al., 2016)
		SHMT2 knockdown (mouse) → tumorigenesis (↓)		
	Colorectal cancer	SHMT2 knockdown (cell) → proliferation (↓), metastasis (↓)	AFAP1-AS1/MLK7-AS1 → miR-149/miR-485 (↓) → SHMT2 (↑)	(Kim et al., 2023)
		SHMT2 knockdown (mouse) → tumor growth (↓), invasion (↓)	Not specified	
		Apigenin treatment (cell) → SHMT2 (↓) → tumor cell apoptosis	G0/G1 phase cell cycle arrest, LINC01234/SIRT3/SHMT2 axis (↓)	(Abdelmaksoud et al., 2024)
		SHMT2 K95Q mutant (cell) → proliferation (↓), tumor growth (↓)	Serine/glycine ratio (↑), NADPH (↓), ROS (↑), and GSH (↓)	(Wei et al., 2018)
		SHMT2 K95Q mutant (mouse) → tumor growth and tumorigenicity (↓)	Not specified	
		SHMT2 knockdown (cell) → proliferation (↓), migration (↓), and invasion (↓)	Ubiquitylation-mediated degradation of β-catenin (↓) TCF4 + β-catenin → SHMT2 expression (↑)	(Liu et al., 2021)
		SHMT2 knockdown (mouse) → tumor growth (↓), metastasis (↓)	Not specified	
		SHMT2 knockdown (cell) → proliferation (↓)	UHRF1(↓), G1/S arrest	(Cui et al., 2022)
		SHMT2 knockdown (mouse) → tumor growth (↓)	UHRF1(↓), Ki-67(↓)	
		Low SHMT2 → 5-FU resistance	p53 and HDM2 binding (↓), p53 degradation (↓), and autophagy (↓)	(Chen et al., 2021)
	Prostate cancer	SHMT2 knockdown (cell) → proliferation (↑), metastasis (↑), migration (↑), and EMT (↑)	ZEB1 (↑), vimentin (↑), N-cadherin (↑), and E-cadherin (↓) p-ERK1/2 (↑)	(Chen et al., 2022)
		SHMT2 knockdown (mouse) → tumor growth (↑)	Not specified	
	Thyroid cancer (ATC, PTC, and PDTC)	SHMT2 knockdown (cell) → proliferation (↓) cell viability (↓), and migration (↓)	Oxygen consumption (↓), OCR (↓), mitochondrial function (↓), and OXPHOS complex proteins (↓)	(Lee et al., 2024b)
		SHMT2 inhibition (cell) → cell growth (↓), mitochondrial function (↓)	M+2 glycine (↓), M+1 serine (↓), M+2 serine (↓), cell viability (↓), mitochondrial respiration (↓), and OXPHOS complex-related protein (complexes I-V) (↓)	
		SHMT2 knockdown or inhibition (mouse) → tumor growth (↓)	Not specified	
	Pancreatic cancer	AGF347 → SHMT2 (↓) → tumor cell proliferation (↓)	AMPK (↑), hypophosphorylation of S6K1, ROS (↑), GSH (↓), and total glutathione (↓)	(Dekhne et al., 2020b)

LUAD, lung adenocarcinoma; NSCLC, non–small-cell lung cancer; SCLC, small-cell lung cancer; AC, pulmonary adenocarcinoma; SQCLC, squamous cell lung cancer; HCC, hepatocellular carcinoma; CRC, colorectal cancer; ATC, anaplastic thyroid carcinoma; PTC, papillary thyroid carcinoma; PDTC, poorly differentiated thyroid carcinoma; HGSOC, high-grade serous ovarian cancer; NADPH, nicotinamide adenine dinucleotide phosphate; ROS, reactive oxygen species; GSH, glutathione; dTMP, deoxythymidine monophosphate; OCR, oxygen consumption rate; ECAR, extracellular acidification rate; AMPK, adenosine monophosphate-activated protein kinase; OCR, oxygen consumption rate; Neu5Ac, sialic acid N-acetylneuraminic acid; OXPHOS, oxidative phosphorylation.

**Table 3:** SHMT role in metabolic disease

Serine hydroxymethyltransferase	Disease type	Model	Role of SHMT	Reference
SHMT1	Metabolic syndrome	Mice and HCC cell line	− SHMT1 → NOX1-mediated ROS production (↓) → EMT and MMP2 expression → HCC cell migration and metastasis.	(Dou et al., 2019)
	Metabolic syndrome	MCF-7 cell line	− SHMT1 → dTMP and SAM competition → regulates folate metabolism. − SHMT1 (high DNA replication demand) → dTMP synthesis, SAM synthesis (↓).	(Herbig et al., 2002)
	Cardiovascular diseases	HAoSMC cell line	− High-phosphate environment → SHMT1 expression (↑). − SHMT1 knockdown → NADPH oxidase components NOX4 and CYBA (↑). − SHMT1 knockdown → MMP2 (↑). − SHMT1 knockdown → BAX/BCL2 (↑), vascular calcification process (↑) → apoptosis.	(Boehme et al., 2018)
SHMT2	Metabolic dysfunction-associated steatotic liver disease (MASLD)	Liver-specific SHMT2 knockout mice	− SHMT2 → regulates glycine levels in the liver, impacts oxidative stress → glutathione synthesis, liver injury. − SHMT2 inhibition or glycine supplementation → restores glutathione and reduces injury.	(Ghrayeb et al., 2024)
		Liver-specific SHMT2 knockout mice	− SHMT2 primarily consumes glycine. − SHMT2 activity fluctuates with glycine levels, dynamically regulating metabolism → prevents excessive or insufficient serum glycine accumulation. − SHMT2 inhibition → circulating glycine (↑), does not ameliorate metabolic syndromes such as fatty liver disease.	(McBride et al., 2024)
	Hepatic lipid metabolism	Liver-specific SHMT2 knockout mice	− SHMT2 → glycine-mediated mTOR/PPARγ pathway activation → modulates lipid accumulation. − SHMT2 knockdown → glycine (↓), mTOR/PPARγ signaling (↓) → lipid accumulation. − Glycine supplementation or mTOR/PPAR activation restores SHMT2-mediated lipid metabolism.	(Choi et al., 2022)
	Liver fibrosis and inflammation	SHMT2 knockout mice	− Early stage: SHMT2 → maintains hepatic methylation balance → protective effect. − Late stage: SHMT2 → SAM-mediated methylation →hepatic inflammation and fibrosis (↑) → liver pathology (↑).	(Chen et al., 2024)
		SHMT2 knockout mice	− SHMT2 is upregulated during liver regeneration. − ↑ SHMT2 → glycine synthesis (↑), Akt/mTOR pathway (↑) → hepatocyte proliferation → liver regeneration (↑). − SHMT2 deficiency → liver-to-body weight ratio (↓), hepatocyte proliferation (↓) → liver regeneration (↓).	(Wang et al., 2019)
	Embryonic anemia	SHMT2-deficient mice	− SHMT2 → maintains OCM, regulates mitochondrial function, cell proliferation, and erythropoiesis → fetal liver development. − SHMT2 loss → mitochondrial OCM in mouse embryos (↓), the differentiation of erythroid progenitors into mature red blood cells (↓) → anemia. − SHMT2 loss → taurine (↓), nucleotide (↓), mitochondrial function (↓), DNA replication (↓), and hepatic metabolism (↓).	(Tani et al., 2019)
	Mitochondrial metabolic imbalance in obesity	Metabolic obesity mice	− SHMT2 → mitochondrial OCM → links energy metabolism with epigenetic regulation. − SHMT2 serves as a key factor in metformin-mediated enhancement of DNA methylation. − In SHMT2 knockout cells, metformin fails to regulate S-adenosylhomocysteine levels or increase DNA methylation.	(Cuyas et al., 2018)
	Obesity	Human	− SHMT2 is upregulated in the muscle tissue of MELAS (mitochondrial encephalomyopathy, lactic acidosis, and stroke-like episodes syndrome) patients → serine catabolism (↑) → energy metabolism dysregulation. − SHMT2 inhibition → excessive NADH accumulation (↓), NAD+/NADH ratio (↑), restores cellular viability.	(Liufu et al., 2024)
	Metabolic syndrome	HepG2 cell line	− SHMT2 depletion → autophagy-lysosome pathway → lysosomal membrane permeabilization and apoptosis. − SHMT2 highly expressed in clear-cell renal cell carcinoma tissues and cells → poor prognosis.	(Liu et al., 2023)

**Table 4:** SHMT role in neurologic disease

Serine hydroxymethyltransferase	Disease type	Model	Role of SHMT	Reference
SHMT1	Neurodevelopmental disorder	SHMT1 knockout mice	− SHMT1 deficiency → the sensitivity of mouse embryos to folate deficiency (↑) → incidence of NTDs (↑). − SHMT1 absence → DNA synthesis (↓), cell proliferation (↓) → normal neural tube development (↓) → NTDs. − SHMT1 inactivation (folate deficiency) → neural tube developmental defects (↑).	(Beaudin et al., 2012, Beaudin et al., 2011)
	Neurodevelopmental disorder	SHMT1 knockout mice	− SHMT1 deficiency → DNA synthesis (↓) → NTDs risk (↑). − SHMT1 loss → dTMP synthesis (↓) → DNA replication (↓) → proper neural tube closure (↓). − SHMT1 knockout mouse (folate deficiency) → NTDs incidence (↑).	(Martiniova et al., 2015)
	Neurodevelopmental disorder	SHMT1 knockout mice	− SHMT1 → regulates hippocampal neurogenesis, affects cognitive function. − SHMT1 → prominently expressed in the cornu ammonis and dentate gyrus regions of the mouse hippocampus (closely associated with memory and learning abilities). − SHMT1 loss → dTMP synthesis (↓) → DNA replication (↓) → neuronal dysfunction (↓).	(Abarinov et al., 2013)
SHMT1	Adult acute lymphoblastic leukemia (ALL)	Human	− SHMT1 C1420T polymorphism → ALL risk (↓). − SHMT1 1420CT heterozygous genotype exhibits a 2.1-fold reduction in the risk of ALL (OR = 0.48, 95% CI: 0.25-0.91). − SHMT1 1420TT homozygous genotype shows a 3.3-fold decrease in ALL risk (OR = 0.31, 95% CI: 0.10-0.90). − SHMT1 mutation → dTMP supply (↑) → uracil misincorporation during DNA replication (↓) → ALL susceptibility (↓).	(Skibola et al., 2002)
SHMT2	Neurodevelopmental disorder	Human genetic mutation studies	− SHMT2 mutation → mitochondrial OCM (↓) → cognitive impairment, microcephaly, and cardiomyopathy. − SHMT2 deficiency → alters glycine/serine balance, DNA replication (↓) → neuronal development.	(Garcia-Cazorla et al., 2020)
	Neurodevelopmental disorder	Human genetic study of a novel missense variant	− Homozygous SHMT2 variant → intellectual disability, spasticity, and peripheral neuropathy. − SHMT2 mutation (Asp378Gly) → protein stability (↓), function (↓) → may contribute to neurological abnormalities and mitochondrial metabolic dysfunction.	(Majethia et al., 2022)
	Alzheimer's disease (AD)	SHMT2 overexpression in AD models	− SHMT2 → mitochondrial OCM (↑) → Alzheimer's pathology (↓), modulating disease-related pathways. − SHMT2 interacting with the GAGGG motif in the 5′UTR of ADAM10 → relieves ADAM10's translational repression → ADAM10 translation (↑) → Aβ deposition (↓), AD pathology (↓), and cognitive function (↑).	(Song et al., 2024)

### SHMT in Lung Cancer

#### SHMT1 in Lung Cancer

Lung cancer cells, particularly those of non–small-cell lung cancer (NSCLC), heavily rely on metabolic reprogramming to sustain rapid proliferation of tumor cells (Chen et al., 2014). Transcriptomic analysis of tumor samples from 97 NSCLC patients revealed significant upregulation of SHMT1 expression under low-folate conditions (Lin et al., 2022). Notably, in NSCLC with concurrent LKB1 and KEAP1 mutations, SHMT1 expression is further upregulated through NRF2-mediated activation, contributing to serine-glycine 1-carbon metabolism and supporting tumor cell survival under oxidative stress (Lee et al., 2024a). Furthermore, metabolomic and transcriptomic analyses of LKB1- and KEAP1-mutant NSCLC cells showed that SHMT1 expression was regulated by NRF2 and MAFK (Lee et al., 2024a). Additionally, plasma proteomic screening results indicated that SHMT1 plays a role in facilitating metabolic adaptation in small-cell lung cancer (SCLC) (Wu et al., 2023).

As a key enzyme in OCM, SHMT1 is critical for nucleotide biosynthesis and cell survival. SHMT1 deficiency leads to a sharp dTMP synthesis, resulting in errors in DNA replication and aberrant uracil incorporation. These disruptions ultimately induce genomic instability and trigger apoptosis of NSCLC cells (Giardina et al., 2018, Paone et al., 2014). Thus, SHMT1 plays a vital role in nucleotide synthesis, genomic stability, and lung cancer cell proliferation. Upregulation of SHMT1 enhances the metastatic capacity of LUAD cells by sustaining high AMPK activity, which is crucial for regulating OCM (Bouzidi et al., 2020).

#### SHMT2 in Lung Cancer

TCGA data showed that SHMT2 mRNA expression was significantly elevated in both lung adenocarcinoma (LUAD) and lung squamous cell carcinoma, as reported in recent studies (Lin et al., 2022, Luo et al., 2021). Further metabolomic and transcriptomic analyses of LKB1- and KEAP1-mutant NSCLC cells revealed that SHMT2 expression was regulated by the NRF2-ATF4 signaling pathway, facilitating serine-glycine 1-carbon metabolism to meet increased demands for 1-carbon units and antioxidant defense (Lee et al., 2024a).

Active primarily in the mitochondria, SHMT2 supports metabolic adaptation under nutrient-deprived conditions. Stable isotope tracing studies have demonstrated that SHMT2 enhances mitochondrial OCM by facilitating serine-to-glycine conversion, enabling cancer cells to survive under metabolic stress (Haitzmann et al., 2024). Furthermore, SHMT2 activity is regulated by the NRF2-ATF4 signaling pathway, with NRF2 knockout leading to reduced ATF4 levels and SHMT2 expression (DeNicola et al., 2015). SHMT2 activity is also regulated post translationally by phosphorylation. MAPK1-mediated phosphorylation at Ser90 markedly enhances SHMT2 stability and enzymatic activity. Phosphorylated SHMT2 promotes SAM production and m6A RNA modification, stabilizing oncogenic mRNAs and facilitating cell proliferation and survival, which ultimately drives LUAD progression (Han et al., 2024).

In addition to its metabolic functions, SHMT2 expression influences the tumor immune microenvironment. TCGA data analysis revealed that high SHMT2 expression was associated with reduced immune cell infiltration, particularly decreased levels of T cells, dendritic cells, and macrophages (Luo et al., 2021). In line with these findings, SHMT2 expression was found to be significantly negatively correlated with CD4+ T cells, macrophages, and dendritic cells, which may facilitate metastasis by fostering an immunosuppressive tumor microenvironment (Luo et al., 2021). Moreover, elevated SHMT2 expression is strongly correlated with poor prognosis in patients with LUAD. Kaplan-Meier survival analysis of clinical tumor samples revealed that patients with adenocarcinoma (AC), squamous cell carcinoma, and SCLC exhibiting high SHMT2 expression had a significantly lower overall survival (OS) than those with low SHMT2 expression (Yao et al., 2021).

This functional distinction between SHMT1 and SHMT2 suggests that targeting both enzymes could disrupt the metabolic reprogramming driving lung cancer progression.

### SHMT in Liver Cancer

#### SHMT1 in Liver Cancer

SHMT1 acts as a tumor suppressor in HCC by suppressing NADPH oxidase 1, thereby reducing ROS levels and inhibiting epithelial-mesenchymal transition (Dou et al., 2019). This suppression also downregulates matrix metalloproteinase 2, ultimately attenuating tumor invasion and metastasis (Dou et al., 2019). Mechanistically, miR-5003-3p has been shown to promote HCC cell metastasis and invasion by directly suppressing SHMT1 expression (Tang et al., 2024).

#### SHMT2 in Liver Cancer

SHMT2 expression is transcriptionally upregulated in HCC cells under extracellular L-serine depletion, suggesting a role in metabolic adaptation to amino acid stress (Hamano et al., 2020). Consistently, high SHMT2 expression correlates with poor differentiation and increased invasion (Ji et al., 2019), while SHMT2 knockdown significantly inhibits HCC cell metastasis and tumor progression (Woo et al., 2016). Moreover, miR-615-5p effectively inhibits the proliferation and metastasis of HCC cells by directly targeting SHMT2 (Wu et al., 2016).

Both SHMT1 and SHMT2 are essential enzymes in the OCM, yet they exert distinct functions in HCC. These differences may be driven by epigenetic mechanisms, such as DNA methylation and microRNA-mediated silencing (Moruzzi et al., 2017). A better understanding of the interactions between SHMT1 and SHMT2 and their regulatory mechanisms in the metabolic network may reveal novel therapeutic vulnerabilities and guide interventions in HCC.

### SHMT in Colorectal Cancer

#### SHMT1 in Colorectal Cancer

Although SHMT1 is known to play important roles in several cancers, including lung cancer and HCC, its biological function and clinical relevance in colorectal cancer (CRC) remain poorly understood.

Animal studies have demonstrated that Shmt1 heterozygosity impairs folate-dependent thymidylate synthesis by downregulating key enzymes such as thymidylate synthase and cytoplasmic thymidine kinase. This impairment increases the risk of intestinal tumor formation in Apc^min/+ mice, indicating that Shmt1 contributes to CRC susceptibility through gene-diet interactions (Macfarlane et al., 2011b). Additionally, another study found that the SHMT1 1420T allele, especially when combined with the MTHFR 677CC genotype, was associated with a reduced risk of rectal cancer but not colon cancer (Komlosi et al., 2010). This genotype combination was also linked to altered plasma homocysteine levels, which may influence carcinogenic mechanisms. These findings suggest that SHMT1 may influence CRC risk through its roles in folate metabolism and genetic variation, although further mechanistic and clinical studies are warranted to validate these associations.

#### SHMT2 in Colorectal Cancer

Elevated SHMT2 expression has been observed at both mRNA and protein levels in CRC tissues (Cui et al., 2022). One study showed that among 60 patients with CRC, 71.7% exhibited high SHMT2 expression (Liu et al., 2019). Kaplan-Meier survival analysis demonstrated that elevated SHMT2 expression was strongly associated with poor recurrence-free survival and disease-specific survival (Liu et al., 2019). SHMT2 was identified as a candidate gene associated with CRC development through integrated GEO data analysis (Wang et al., 2022). High SHMT2 expression is also strongly correlated with advanced CRC TNM stages (III and IV) and lymph node metastasis in CRC patients (Cui et al., 2022, Liu et al., 2019).

SHMT2 is crucial for the rapid proliferation of CRC cells as it catalyzes the conversion of serine to glycine to drive the OCM pathway. The natural compound apigenin downregulates SHMT2 by targeting the LINC01234/SIRT3/SHMT2 axis to suppress metabolic reprogramming in CRC cells, disrupt cell cycle progression, and induce apoptosis through the downregulation of SHMT2 (Abdelmaksoud et al., 2024). Further research revealed that SIRT3 maintains SHMT2 enzymatic activity and protein stability by deacetylating lysine 95 (K95). This regulation enhances NADPH production, supports antioxidant defense, and preserves cellular redox balance in CRC (Wei et al., 2018).

In addition to its metabolic roles, SHMT2 also plays an important role in nonmetabolic functions that are essential for CRC cell proliferation. Mechanistically, SHMT2 stabilizes β-catenin by preventing its ubiquitin-mediated degradation, promoting nuclear accumulation and activation of oncogenic targets such as c-Myc and CD44. In turn, β-catenin binds to TCF4 to upregulate SHMT2 transcription, establishing a positive feedback loop that drives both proliferation and metastasis in CRC (Liu et al., 2021). Moreover, SHMT2 alters DNA methylation patterns, thereby regulating the expression of tumor-related genes, highlighting its role in the epigenetic regulation of CRC progression (Wang et al., 2022). Additional studies showed that the antisense RNAs AFAP1-AS1 and MLK7-AS1 indirectly increased SHMT2 expression by sponging miR-149-5p and miR-485-5p, respectively. This upregulation enhanced the proliferation, migration, and metastatic capacities of CRC cells (Kim et al., 2023).

### SHMT in Prostate Cancer

#### SHMT1 in Prostate Cancer

In prostate cancer, SHMT1 has been implicated in metabolic rewiring and may be involved in coordinating 1-carbon flux alongside other metabolic enzymes. An analysis of TCGA prostate cancer cohort (n = 498) reported SHMT1 dysregulation in approximately 5% of tumors, mainly through elevated mRNA expression. Coexpression network analysis revealed that SHMT1 was significantly coexpressed with other OCM genes such as MTHFD1 and PSAT1, suggesting its potential role in maintaining metabolic homeostasis in tumor cells (Ganini et al., 2021). To date, no direct mechanistic studies have been conducted to assess the impact of SHMT1 on tumor growth, survival, or clinical outcomes in prostate cancer. Further research is warranted to clarify whether SHMT1 serves as a functional driver or passive marker in prostate tumorigenesis.

#### SHMT2 in Prostate Cancer

SHMT2 is highly expressed in early-stage prostate cancer compared with benign prostate tissue (Chen et al., 2022). A study showed that high SHMT2 was associated with shorter disease-free survival (DFS) (Sant’Anna-Silva et al., 2021). GEO data also showed that high SHMT2 levels were correlated with poor survival (Lyu et al., 2017). Mechanistically, SHMT2 was regulated by the IL-6/STAT3 signaling pathway in LNCaP cells. SHMT2 and STAT3 jointly regulate prostate cancer metabolism and promote tumor progression (Marrocco et al., 2019). Research also revealed that the metabolite succinate enhanced tumor metabolic adaptation by upregulating SHMT2 expression, thereby supporting rapid tumor growth (Sant’Anna-Silva et al., 2021). Additionally, SHMT2 synergizes with mitochondrial proteases LONP1 and ClP to maintain mitochondrial protein homeostasis and enhance cancer cell survival (Lee et al., 2021).

However, the role of SHMT2 in metastasis appears to be context- and stage-dependent. Elevated SHMT2 expression has been observed in prostate tumors with high Gleason scores, suggesting an association with more aggressive stages of prostate cancer (Marrocco et al., 2019). Further research has indicated that TWIST, an important transcription factor closely associated with tumor invasiveness and metastasis, may upregulate SHMT2 expression, driving tumor invasion and metastasis (Lyu et al., 2017). Conversely, another study reported high SHMT2 expression in early-stage prostate cancer, with significant downregulation in aggressive late-stage tumors (Chen et al., 2022). Interestingly, this downregulation promoted prostate cancer proliferation and metastasis through activation of the ERK pathway and induction of epithelial-mesenchymal transition (Chen et al., 2022).

Collectively, these studies suggest that SHMT2 may exert stage-specific effects on prostate cancer progression and metastasis, while the role of SHMT1 in this process remains largely unexplored.

### SHMT in Thyroid Cancer

#### SHMT1 in Thyroid Cancer

SHMT1 exhibits subtype-specific expression patterns in thyroid cancer. Immunohistochemical analysis of 557 tumor samples revealed that SHMT1 expression was significantly elevated in PTC and poorly differentiated thyroid carcinoma, but was lowest in medullary thyroid carcinoma (Sun et al., 2016). High SHMT1 expression was observed predominantly in conventional PTC and was associated with the presence of the BRAF V600E mutation, increased invasiveness, and poor DFS (Sun et al., 2016). In contrast, SHMT1 expression was low or absent in follicular and medullary thyroid carcinomas (Sun et al., 2016).

A recent multiomics study integrating single-cell and bulk RNA sequencing with metabolomics revealed that SHMT1 expression declined with decreasing tumor differentiation, with significantly lower levels in anaplastic thyroid carcinoma (ATC) compared with PTC or normal tissue (Lee et al., 2024b). This trend was consistent across bulk and single-cell transcriptomic data and may reflect a metabolic shift from cytosolic to mitochondrial OCM as cancer progresses. While SHMT1 may play a role in maintaining the differentiated state of thyroid cells, its precise function in thyroid tumorigenesis and therapeutic relevance remains to be elucidated.

#### SHMT2 in Thyroid Cancer

SHMT2 plays a distinct and pivotal role in thyroid cancer progression, particularly in ATC, where its expression is significantly upregulated compared with PTC and normal thyroid tissue (Lee et al., 2024b). Notably, SHMT2 expression increased as tumor cells became less differentiated and was inversely correlated with the thyroid differentiation score (Lee et al., 2024b). Genes involved in mitochondrial OCM, including SHMT2, were enriched in ATC cells, suggesting a metabolic reprogramming that distinguishes them from more differentiated subtypes (Lee et al., 2024b).

Furthermore, knockout or pharmacological inhibition of SHMT2 significantly suppressed the proliferation and migration of ATC cell lines, including 8505C and FRO, compared with PTC cell lines (Lee et al., 2024b). When SHMT2-knockdown cells or cells treated with an SHMT2 inhibitor, SHIN2, were injected into mice, the resulting xenograft tumors were markedly smaller. Tumors also exhibited lower Ki-67 staining, indicating reduced proliferative activity and corroborating the observed tumor volume reduction (Lee et al., 2024b).

Collectively, these findings identify mitochondrial SHMT2 as a central driver of metabolic remodeling and tumor aggressiveness in ATC. Given its distinct expression pattern and essential function in undifferentiated thyroid tumors, SHMT2 represents a promising therapeutic target, particularly for ATC, which currently lacks effective treatment options.

### SHMT in Pancreatic Cancer

#### SHMT1 in Pancreatic Cancer

Compared with SHMT2, the biological role of SHMT1 in pancreatic cancer remains poorly understood. A study has shown that the L474F single-nucleotide polymorphism in SHMT1 is significantly associated with folate levels but has no significant association with pancreatic cancer risk (Chittiboyina et al., 2018). Although the L474F site of SHMT1 was not confirmed to have a direct protective effect against pancreatic cancer, the study suggested that the loss of SHMT1 nuclear localization may be compensated by SHMT2 through metabolic mechanisms, thereby playing a role in the tumor environment (Chittiboyina et al., 2018). To date, however, no clear evidence has demonstrated a functional tumor-promoting or tumor-suppressive role for SHMT1 in PC, highlighting the need for further investigation.

#### SHMT2 in Pancreatic Cancer

SHMT2 is significantly upregulated in pancreatic ductal adenocarcinoma tissues and is localized in the cytoplasm of tumor cells (Noguchi et al., 2018). Immunohistochemical analyses of 103 pancreatic ductal adenocarcinoma samples revealed that high SHMT2 expression was significantly associated with poor OS but not with DFS (Noguchi et al., 2018). In univariate and multivariate Cox regression analyses, SHMT2 was identified as an independent prognostic factor for OS (*P* = .017), supporting its clinical relevance (Noguchi et al., 2018). Notably, patients with high expression of SHMT2, along with other mitochondrial folate enzymes such as methylenetetrahydrofolate dehydrogenase 2 and ALDH1L2, exhibited the poorest survival outcomes, while triple-low expression was associated with significantly improved OS and DFS (Noguchi et al., 2018). These findings indicate that SHMT2 contributes to the malignant phenotype of pancreatic cancer and may serve as a potential therapeutic target.

### SHMT in Ovarian Cancer

#### SHMT1 in Ovarian Cancer

Ovarian cancer, particularly high-grade serous ovarian cancer (HGSOC), is a lethal gynecological malignancy (Kroeger and Drapkin, 2017). The metabolic requirements of HGSOC are still poorly understood; however, tumor metabolic reprogramming is considered an important feature of cancer cell survival, proliferation, and metastasis (Lee and Yoon, 2024). Comprehensive gene expression analysis of HGSOC patient samples revealed that SHMT1 expression is significantly elevated in ovarian cancer. SHMT1 knockdown has the potential to inhibit PEO4 cell growth, and inhibits the colony-forming ability of other ovarian cancer cell lines, COV504 and COV413B, in soft agar. Similarly, the loss of SHMT1 inhibited ovarian tumor growth in vivo (Gupta et al., 2017). In ovarian cancer, SHMT1 not only promotes tumor growth but also facilitates metastasis. SHMT1 knockout significantly reduced colony formation on soft agar and diminished the migratory capacity of ovarian cancer cells in vitro (Gupta et al., 2017). Mechanistically, SHMT1 promotes ovarian cancer metastasis and progression by increasing Neu5Ac levels and upregulating the proinflammatory cytokines including IL-6 and IL-8 (Gupta et al., 2017).

#### SHMT2 in Ovarian Cancer

Analysis of RNA-sequencing data from TCGA revealed that SHMT2 expression is elevated in ovarian cancer tissues, and high SHMT2 levels are associated with poor prognosis in patients (Wallace-Povirk et al., 2024, Zeng et al., 2021). These observations suggest that SHMT2 may serve as a prognostic biomarker and a potential therapeutic target in ovarian cancer.

In addition to expression level, SHMT2 genetic polymorphisms may also influence ovarian cancer susceptibility. A case-control study indicated that certain single-nucleotide polymorphisms in the SHMT2 gene, particularly in combination with dietary or genetic factors such as folate status or MTHFR genotype, were associated with altered risk of ovarian carcinoma (Kelemen et al., 2012).

Moreover, recent findings have uncovered an association between SHMT2 isoform switching and chemoresistance in ovarian cancer. Cisplatin-resistant and -sensitive ovarian cancer cells express distinct SHMT2 isoforms, regulated by alternative promoter usage. Specifically, isoform 1 appears to suppress cancer stem-like properties, while isoform 3 promotes them. Transcription factors such as HIF1α and TFE3 mediate isoform expression in response to metabolic stress, including hypoxia and glucose deprivation, providing a mechanistic link between SHMT2 isoform regulation and cisplatin resistance (Wang et al., 2025).

Together, these findings suggest that SHMT2 contributes to ovarian cancer development, prognosis, and therapeutic response through its expression level, genetic variants, and isoform-specific regulatory mechanisms.

## ROLE OF SHMT IN CANCER TREATMENT RESISTANCE

5-Fluorouracil (5-FU), an antimetabolite chemotherapeutic agent, is widely used to treat CRC, pancreatic cancer, and breast cancer (Vodenkova et al., 2020, Wang et al., 2014, Fahmy et al., 2024). In CRC, SHMT2 has been implicated in 5-FU resistance through multiple mechanisms. On one hand, SHMT2 contributed to 5-FU resistance by increasing nucleotide biosynthesis and facilitating DNA damage repair (Pranzini et al., 2022). On the other hand, loss of SHMT2 can also contribute to chemoresistance by inducing autophagy and inhibiting apoptosis in CRC cells (Chen et al., 2021), suggesting that both overexpression and depletion of SHMT2 may promote cell survival under chemotherapy, albeit via distinct pathways. Doxorubicin is a commonly used chemotherapeutic agent (Rivankar, 2014). In HCC, the downregulation of SHMT2 increases the sensitivity of cancer cells to doxorubicin, suggesting that SHMT2 inhibition can overcome treatment resistance in HCC (Woo et al., 2016). Cisplatin is a widely used anticancer chemotherapeutic agent (Dasari and Tchounwou, 2014). In ovarian cancer, elevated SHMT2 expression is linked to cisplatin resistance, by which it enhances the DNA repair capacity and metabolic adaptability, thereby promoting cell survival under chemotherapeutic stress (Wallace-Povirk et al., 2024). AGF347, a pyridopyrimidine-based SHMT2 inhibitor, has demonstrated antitumor activity (Dekhne et al., 2020b). A previous study revealed that AGF347 effectively reduced tumor volume in a mouse model of pancreatic cancer by targeting SHMT2 (Nayeen et al., 2023). Another study showed that targeting SHMT2 with AGF347 effectively inhibited the proliferation of several pancreatic cancer cell lines, including MIA PaCa-2, HPAC, and BxPC-3 (Dekhne et al., 2020b). Notably, the antitumor efficacy of AGF347 exceeded that of the conventional chemotherapeutic agent gemcitabine (Dekhne et al., 2019). However, the efficacy of AGF347 may be influenced by folate polyglutamate levels, which regulate its accumulation and anticancer activity in pancreatic cancer cells (O'Connor et al., 2024). S-1 is an oral anticancer drug that combines tegafur, gimeracil, and oteracil to enhance therapeutic efficacy while minimizing side effects (Abdel-Rahman and ElHalawani, 2016). It is widely used to treat NSCLC, CRC, and other cancers. S-1 significantly downregulates SHMT1 expression, which is correlated with increased drug sensitivity in NSCLC cells (Matsuoka et al., 2015). A study demonstrated that nuclear glycogen synthase kinase 3 (GSK3) suppresses SHMT2 expression, thereby limiting serine/one-carbon metabolism in lung cancer cells. Inhibition of GSK3 leads to upregulation of SHMT2, resulting in a metabolic dependency that can be exploited using SHMT inhibitors. Accordingly, GSK3 and SHMT dual inhibition showed synergistic antitumor effects in preclinical models, providing a promising therapeutic strategy (He et al., 2022). Additionally, combining SHMT inhibitors with paclitaxel significantly suppressed KRAS/LKB1/KEAP1-mutant (KLK) NSCLC growth and enhanced chemosensitivity (Lee et al., 2024a).

Collectively, although both SHMT1 and SHMT2 participate in OCM, recent studies have highlighted SHMT2 as a key contributor to drug resistance, possibly due to its mitochondrial localization and elevated expression in various tumors (Chen et al., 2021, He et al., 2022). In contrast, the role of SHMT1 in chemoresistance remains less well-defined and warrants further investigation (Matsuoka et al., 2015).

## FUTURE PERSPECTIVES AND THERAPEUTIC IMPLICATIONS

SHMT, especially SHMT2, has emerged as a promising therapeutic target in diverse cancers. Its roles in nucleotide synthesis and redox homeostasis are critical for tumor cell survival. Inhibition or knockdown of SHMT isoforms has been shown to suppress proliferation and metastasis in multiple cancers, including lung cancer (Lee et al., 2024a), liver cancer (Dou et al., 2019), and thyroid cancer (Lee et al., 2024b). These findings suggest SHMT may represent a metabolic vulnerability across multiple malignancies.

**Fig. 2:**
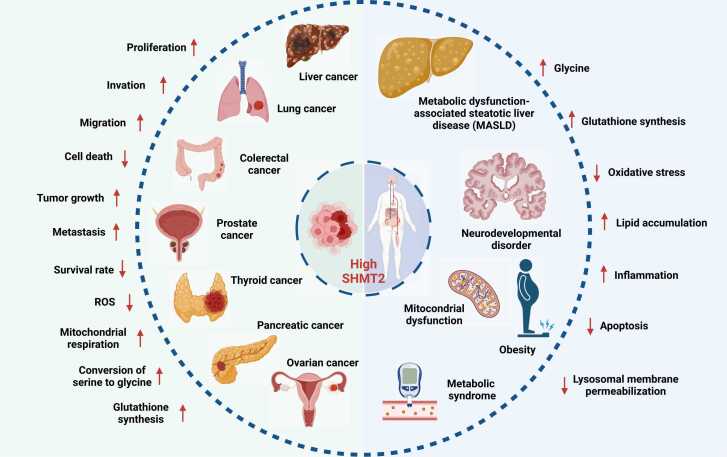
Overview of SHMT-associated roles in cancer and metabolic disorders. Illustrates the biological role of high SHMT expression in cancer and metabolic disorders, highlighting its dual function in promoting tumorigenesis and impacting systemic metabolism. Elevated SHMT levels are linked to enhanced tumor cell proliferation, invasion, migration, growth, and metastasis, while simultaneously reducing cell death and survival rates. SHMT contributes to increased mitochondrial respiration, reactive oxygen species (ROS) production, conversion of serine to glycine, and glutathione synthesis in tumors such as lung, liver, colorectal, prostate, thyroid, pancreatic, and ovarian cancers being notably affected. Beyond cancer, SHMT dysregulation drives noncancerous conditions, including metabolic dysfunction-associated steatotic liver disease (MASLD), which is characterized by lipid accumulation and inflammation, as well as neurodevelopmental disorders linked to mitochondrial dysfunction and altered metabolism. It also disrupts mitochondrial energy production, contributing to oxidative stress, obesity, metabolic syndromes, inflammation, and lipid dysregulation. Furthermore, SHMT influences apoptosis and lysosomal membrane permeabilization, thereby impairing cellular homeostasis. At the systemic level, SHMT promotes metabolic changes, such as increased glycine levels, enhanced glutathione synthesis, and lipid accumulation, while mitigating oxidative stress during inflammation. These findings highlight the pivotal role of SHMT as a metabolic regulator of cancer progression and systemic metabolic dysfunction.

Future strategies may involve isoform-specific inhibitors such as SHIN2, or combination therapies targeting SHMT alongside folate metabolism or immune pathways. Beyond oncology, SHMT modulation may also have therapeutic potential in metabolic and cardiovascular diseases where redox imbalance plays a role (Fig. 2). However, further research is needed to define optimal therapeutic windows and assess long-term safety.

## CONCLUSION

SHMT1 and SHMT2, which are key enzymes in OCM, play distinct yet interconnected roles in cancer progression and metabolic disorders. SHMT1 primarily functions in the cytoplasm and regulates DNA synthesis, methylation, and redox homeostasis, whereas SHMT2 is predominantly mitochondrial and contributes to serine-glycine metabolism, oxidative stress balance, and metabolic adaptation. In cancer, SHMT1 sometimes acts as a tumor suppressor, modulating redox homeostasis and inhibiting metastasis, whereas SHMT2 often supports tumor proliferation and survival by enhancing nucleotide biosynthesis and metabolic flexibility. In contrast, SHMT1 influences lipid metabolism through DNA methylation and adipocyte differentiation in metabolic diseases such as obesity and metabolic syndrome, whereas SHMT2 regulates mitochondrial function, glycine metabolism, and oxidative stress resistance (Table 1, Table 2, Table 3, Table 4).

Given their distinct metabolic functions, targeting SHMT1 and SHMT2 is a promising therapeutic strategy for cancer and metabolic diseases. Future studies should focus on unraveling the regulatory mechanisms, identifying disease-specific metabolic vulnerabilities, and optimizing SHMT-targeted therapies to advance precision medicine in oncology and metabolic health.

## FUNDING AND SUPPORT

This work was supported by National Research Foundation of Korea (NRF) grants funded by the Korean government (MSIT) (grant numbers RS-2021-NR061617 to YEK, RS-2022-NR071878 to DHK, and RS-2025-00558025 to SEL), the Basic Science Research Program through the NRF funded by the Ministry of Education (grant number RS-2024-00463129), grants from the Korea Health Technology R&D Project through the Korea Health Industry Development Institute (KHIDI), funded by the Ministry of Health & Welfare, Republic of Korea (grant numbers RS-2020-KH088690 and RS-2022-KH130308), the National Research Foundation of Korea (NRF) (grant number RS-2024-00406568) funded by the Korean government (MSIT), and the Korean Thyroid Association Clinical Research Award 2021.

## CRediT authorship contribution statement

**Bon Jeong Ku:** Writing – review and editing, Investigation. **Junyoung O. Park:** Writing – review and editing, Supervision. **Seong Eun Lee:** Writing – original draft, Conceptualization. **Jiyeon Yoon:** Methodology, Investigation. **Da Hyun Kang:** Writing – review and editing, Writing – original draft, Conceptualization. **Jun Young Heo:** Writing – review and editing, Supervision. **Yea Eun Kang:** Writing – review and editing, Writing – original draft, Supervision, Conceptualization. **Jing Zhang:** Writing – original draft, Visualization, Investigation, Data curation.

## DECLARATION OF COMPETING INTERESTS

The authors declare that they have no known competing financial interests or personal relationships that could have appeared to influence the work reported in this paper. The authors declare no competing interests.

## Data Availability

This study does not report any new data. All data presented in this review are derived from published literature and publicly available sources.
